# The role of deep learning in diagnostic imaging of spondyloarthropathies: a systematic review

**DOI:** 10.1007/s00330-024-11261-x

**Published:** 2024-12-10

**Authors:** Mahmud Omar, Abdulla Watad, Dennis McGonagle, Shelly Soffer, Benjamin S. Glicksberg, Girish N. Nadkarni, Eyal Klang

**Affiliations:** 1 Tel-Aviv University, Faculty of Medicine, Tel-Aviv, Israel; 2 Department of Medicine B and Zabludowicz Center for Autoimmune Diseases, Sheba Medical Center, Tel-Hashomer, Ramat-Gan, Israel; 3 Section of Musculoskeletal Disease, NIHR Leeds Musculoskeletal Biomedical Research Centre, Leeds Institute of Rheumatic and Musculoskeletal Medicine, University of Leeds, Chapel Allerton Hospital, Leeds, UK; 4 Institute of Hematology, Davidoff Cancer Center, Rabin Medical Center, Petah-Tikva, Israel; 5 The Charles Bronfman Institute of Personalized Medicine, Icahn School of Medicine at Mount Sinai, New York, New York, USA; 6 Division of Data-Driven and Digital Medicine (D3M), Icahn School of Medicine at Mount Sinai, New York, New York, USA

**Keywords:** Spondyloarthropathies, Deep learning, Diagnostic imaging, Convolutional neural networks, Sacroiliitis

## Abstract

**Aim:**

Diagnostic imaging is an integral part of identifying spondyloarthropathies (SpA), yet the interpretation of these images can be challenging. This review evaluated the use of deep learning models to enhance the diagnostic accuracy of SpA imaging.

**Methods:**

Following PRISMA guidelines, we systematically searched major databases up to February 2024, focusing on studies that applied deep learning to SpA imaging. Performance metrics, model types, and diagnostic tasks were extracted and analyzed. Study quality was assessed using QUADAS-2.

**Results:**

We analyzed 21 studies employing deep learning in SpA imaging diagnosis across MRI, CT, and X-ray modalities. These models, particularly advanced CNNs and U-Nets, demonstrated high accuracy in diagnosing SpA, differentiating arthritis forms, and assessing disease progression. Performance metrics frequently surpassed traditional methods, with some models achieving AUCs up to 0.98 and matching expert radiologist performance.

**Conclusion:**

This systematic review underscores the effectiveness of deep learning in SpA imaging diagnostics across MRI, CT, and X-ray modalities. The studies reviewed demonstrated high diagnostic accuracy. However, the presence of small sample sizes in some studies highlights the need for more extensive datasets and further prospective and external validation to enhance the generalizability of these AI models.

**Key Points:**

***Question*** *How can deep learning models improve diagnostic accuracy in imaging for spondyloarthropathies (SpA), addressing challenges in early detection and differentiation from other forms of arthritis?*

***Findings*** *Deep learning models, especially CNNs and U-Nets, showed high accuracy in SpA imaging across MRI, CT, and X-ray, often matching or surpassing expert radiologists.*

***Clinical relevance*** *Deep learning models can enhance diagnostic precision in SpA imaging, potentially reducing diagnostic delays and improving treatment decisions, but further validation on larger datasets is required for clinical integration.*

## Introduction

Spondyloarthropathies (SpA) are a group of inflammatory rheumatic diseases that primarily affect the spine and sacroiliac joints [1]. These conditions, including ankylosing spondylitis and psoriatic arthritis, can cause chronic pain and disability [1]. Diagnosis of SpA is often challenging due to its diverse clinical presentations and gradual onset of symptoms [1–3]. This complexity contributes to a significant diagnostic delay, typically ranging from 7 to 10 years [2, 3].

Medical imaging, including X-ray, magnetic resonance imaging (MRI), and computed tomography (CT), plays a crucial role in the diagnosis and management of SpA [1]. X-rays are commonly used for screening, particularly to detect structural changes in advanced stages [1–3]. MRI is the preferred modality for early diagnosis, as it can detect inflammation and soft tissue changes that may not be visible on X-rays [1–3]. CT is less commonly used but can provide detailed visualization of bony structures when needed [1–3]. However, interpreting these images is sometimes difficult due to the subtle and variable manifestations of SpA [2, 4]. Radiographic changes may be minimal in the early stages, and MRI findings can be non-specific [2, 4, 5]. These challenges in clinical and radiologic diagnosis underscore the need for improved diagnostic tools and strategies.

Recent advancements in artificial intelligence (AI), specifically deep learning, have shown promise in enhancing diagnostic accuracy across various medical fields, including radiology and rheumatology [6–9]. Deep learning employs artificial neural networks designed to analyze images efficiently. Convolutional Neural Networks (CNNs), a deep learning method, have improved image analysis by automatically extracting features from radiological images, allowing for more accurate and efficient interpretation [10–14].

In this systematic literature review, we assess the effectiveness of deep learning techniques in enhancing the accuracy of diagnostic imaging for SpA. Our goal is to determine how these advanced models improve the precision of imaging interpretations in a clinical setting. We will use established performance metrics such as sensitivity and specificity, assess the models’ ability to detect and classify different types of SpA-related changes and look for comparisons of their performances directly with expert radiologists.

## Fundamental concepts of deep learning and computer vision

### Computer vision tasks in medical imaging

Computer vision is a field of artificial intelligence that trains computers to interpret and understand visual information from the world [15]. In medical imaging, Deep Learning models are often applied to Computer Vision tasks to analyze and interpret images [13, 15] (Fig. S1 in the supplementary materials). Three essential computer vision tasks—classification, detection, and segmentation—use deep learning algorithms to improve diagnostic precision [16, 17].

Classification assigns each image to a specific category based on established criteria. In the context of SpA, deep learning models can distinguish between normal, inflammatory, and structural changes. This task is fundamental for assessing disease stage, guiding treatment decisions, and evaluating treatment effectiveness [16, 17].

Detection involves locating key features in medical images and marking them with a region of interest [13]. For SpA, this task may target the identification of inflammation or structural changes such as erosions or fusions in the spine and sacroiliac joints. Deep learning algorithms scan images and visually mark significant abnormalities, aiding clinicians in quickly identifying critical areas [16, 17].

Segmentation partitions a digital image into segments by delineating the exact pixel-wise borders of areas of interest, such as lesions or organs. In SpA, segmentation may accurately outline affected areas in the joints or spine, thereby enabling measurement of the extent of inflammation or bone growth. This precision is essential for assessing disease severity and monitoring progression [13, 16, 17].

### Deep learning models in medical imaging

Currently, in the computer vision field, the primarily used deep learning algorithm is convolutional neural networks (CNN) [9, 18]. This methodology excels in image analysis by identifying repeating patterns through a multi-layered approach [18] (Fig. S2 in the supplementary materials). U-Net, a subset of CNN, specializes in segmenting medical images to precisely highlight areas of interest [19], such as borders of an inflammatory process [20]. CNNs include several types of models, such as ResNet, EfficientNet, and others. These models can analyze intricate details in images, including complex 3-dimensional, multi-anatomical planes (Fig. S3 in the supplementary materials) [21–24].

These models have transformed radiology, enhancing diagnostic accuracy and efficiency in interpreting a wide range of imaging modalities [6, 19, 21, 25–27]. These techniques have also proven effective in detecting subtle signs of SpA, differentiating it from other conditions, and aiding in the assessment of disease progression [10, 11, 24, 28].

## Methods

### Search strategy

We registered this review with PROSPERO (International Prospective Register of Systematic Reviews; CRD42024517372) and followed PRISMA (Preferred Reporting Items for Systematic Reviews and Meta-Analyses) guidelines [29, 30]. We searched PubMed, Embase, Web of Science, and Scopus from their inception until February 2024. We also screened reference lists and searched Google Scholar. Our search terms included “artificial intelligence”, “deep learning”, “spondyloarthritis”, and imaging modalities. Full search strings are available in the Supplementary Materials.

### Study selection

We included original research articles that focused on the integration of deep learning in the radiographic diagnosis of SpA. Studies were selected if they provided data for assessing the performance metrics of models, such as area under the curve, accuracy, sensitivity, and specificity.

We excluded review papers, case reports, conference abstracts, editorials, preprints, and studies not conducted in English.

### Data extraction

Two independent reviewers (M.O. and E.K.) extracted data from each study using a structured form, ensuring coverage of relevant variables. These included the title, author, publication year, study design, radiology method used, the body part examined, research task, sample size, AI method/model employed, performance metrics, limitations, main results, and their implications. Any differences in data extraction were resolved through collaborative discussion, with a third reviewer’s input sought when necessary.

### Risk of bias

To evaluate the quality and robustness of the methodologies in the included studies, the QUADAS-2 (Quality Assessment of Diagnostic Accuracy Studies-2) tool was used [31].

## Results

### Search results and study selection

**Fig. 1:**
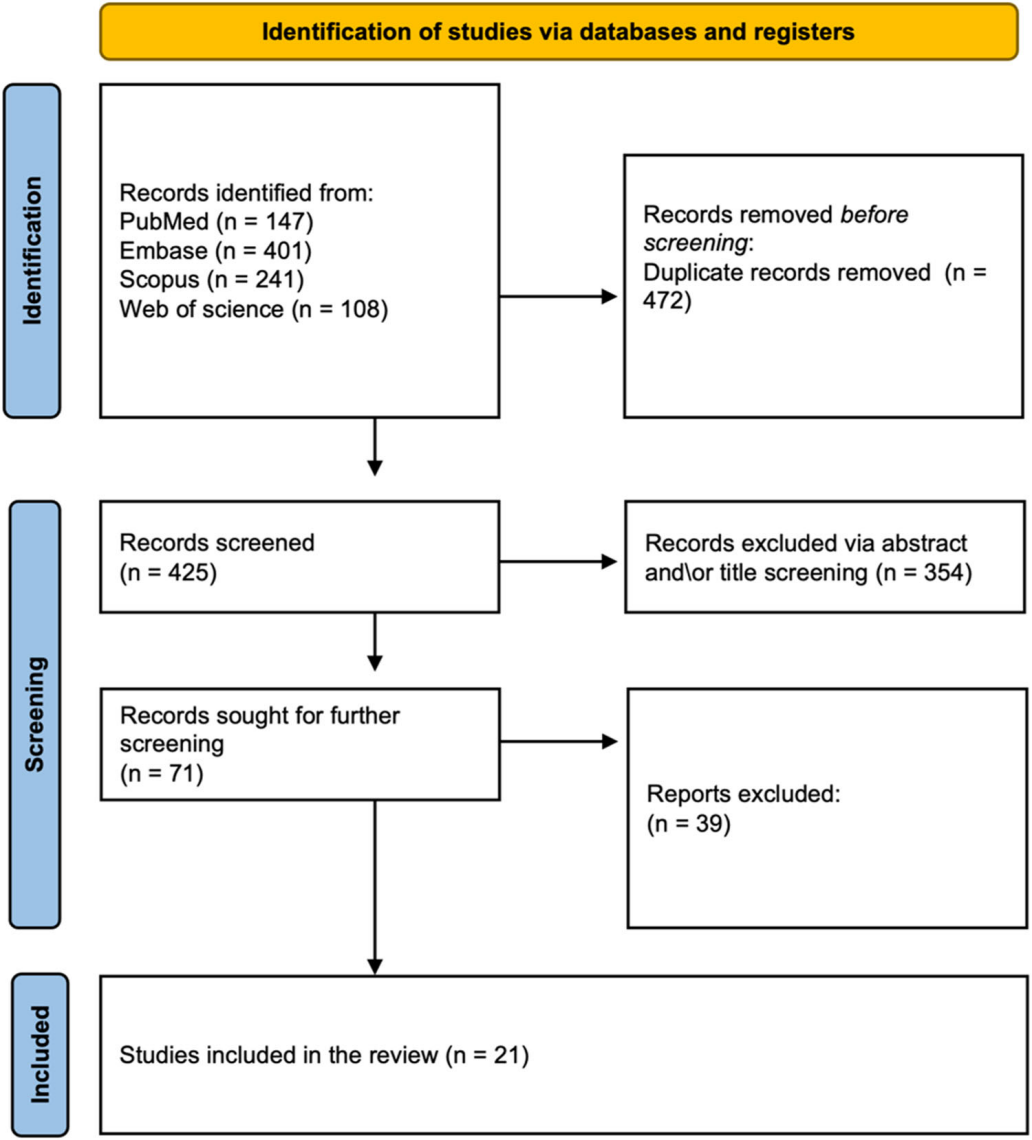
PRISMA flowchart

Our search across PubMed, Embase, Web of Science, and Scopus initially identified 897 papers. After removing 472 duplicates, 425 articles remained. Subsequent title and abstract screening excluded 354 papers, leaving 71 full-text articles for evaluation. Ultimately 21 articles were selected for inclusion. The selection process is visually represented in Fig. 1, the PRISMA flowchart.

#### Risk of bias

**Fig. 2:**
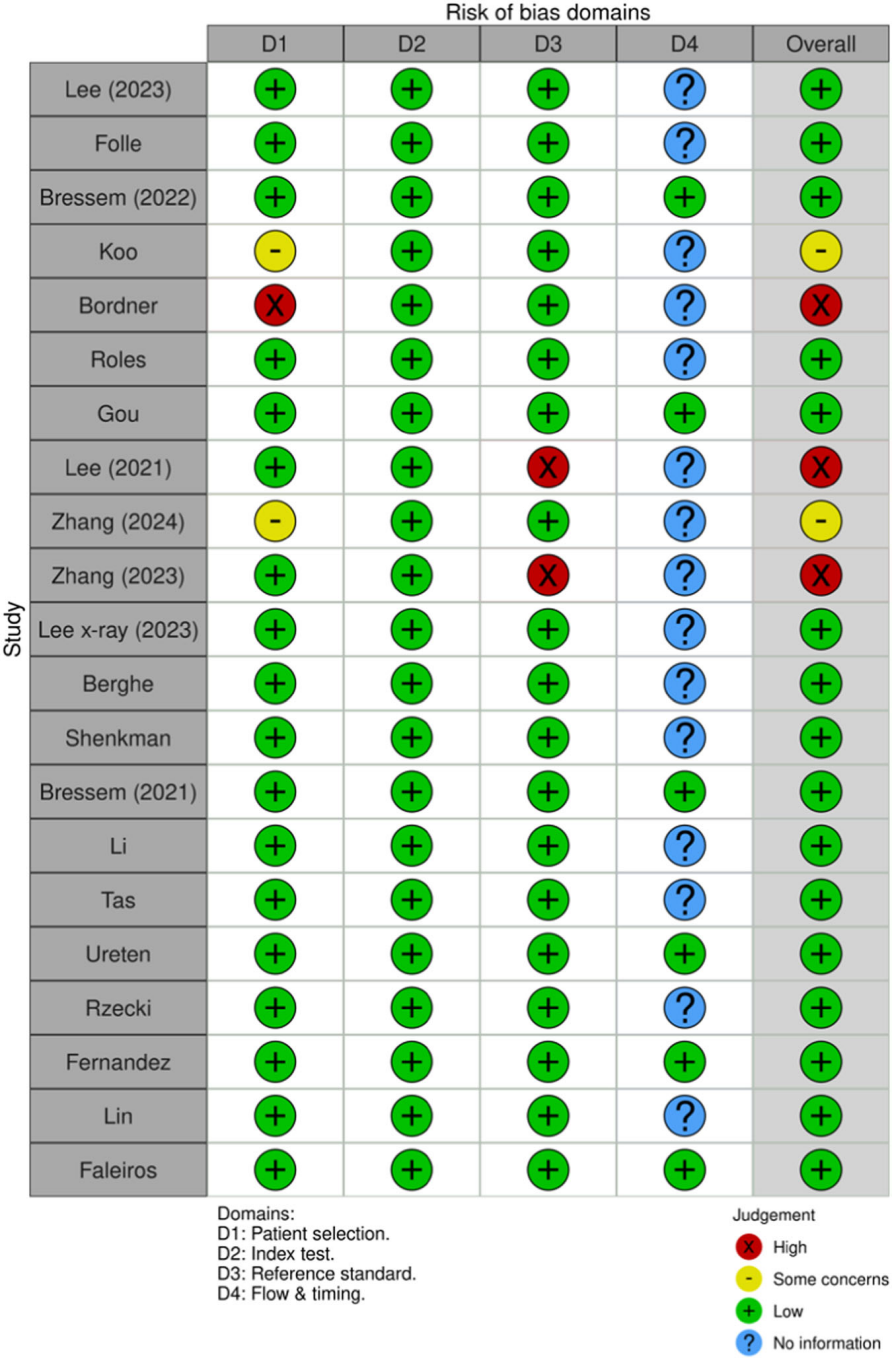
Distribution of risk of bias concerns across individual domains

**Fig. 3:**
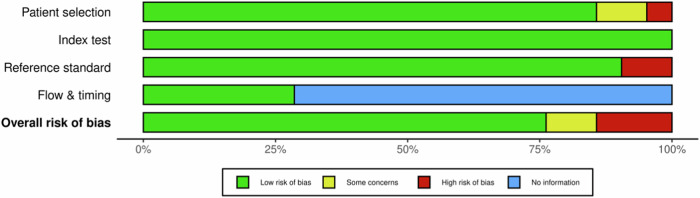
Cumulative assessment of overall risk of bias concerns across all included studies

The risk of bias analysis highlighted that most studies demonstrated a low risk across various domains. Specifically, seven studies exhibited low risk in all evaluated domains, reinforcing their credibility and methodological robustness [11, 24, 32, 34, 36, 41, 45]. Overall, 16 studies were classified as low risk [10, 11, 22–24, 28,32–39, 41, 42, 45], three as high risk [20, 40, 43], and two as presenting some concerns [12, 44] (Figs. 2, 3), underscoring a generally low risk of bias. Regarding applicability concerns, most studies showcased a low risk, indicating their relevance to broader clinical settings. However, it is noteworthy that some studies faced limitations due to small sample sizes.

#### Overview of included studies

This systematic review includes 21 studies exploring the use of deep learning in SpA imaging diagnosis, published between 2019 and 2024 [6–9, 15, 17–20, 26–38] (Fig. S3). These studies were predominantly published in impactful quartile 1 (Q1) journals assessed by SCImago Journal Rank (Fig. S4, Table S1 in the supplementary materials).

The studies involved diverse patient populations. Sample sizes range from smaller cohorts, such as 56 patients in Faleiros et al [32], to more extensive datasets, like the 6436 pelvic X-rays analyzed in Li et al [10]. The clinical tasks implemented were also diverse, addressing different aspects of SpA diagnosis and management. Examples include the detection of inflammatory sacroiliitis [22, 43], differentiating arthritis types [23], grading vertebral changes [44], and predicting the course of ankylosing spondylitis [10] (Table 1).

**Table 1:** A summary of the included studies

Author	Year	Sample size (patients/images)	Radiology method	AI model	Clinical task	Performance metrics
Zhang [9]	2024	485 patients	MRI	ResNet-50, ResNet-101, DenseNet121	Diagnosing sacroiliitis in axSpA	AUC: 0.839, Accuracy: 0.804
Lin [27]	2024	330 patients	MRI	Attention U-Net algorithm	Detecting spinal inflammation in axSpA	Sensitivity: 0.80, Specificity: 0.88
Lee [17]	2023	296/4746 MRI slices	MRI	Faster R-CNN, VGG-19	Detection of inflammatory sacroiliitis	Sensitivity: 0.725, Specificity: 0.936, AUC: 0.830
Bordner [36]	2023	256/362 MRI exams	MRI	Mask-RCNN	Predicting active sacroiliitis	MCC: 0.90, AUC: 0.98
Roles [35]	2023	279/243 patients	MRI	ResNet18-based CNN	Predicting bone marrow edema	Cross-validation AUC: 94.5%, Balanced Accuracy: 80.5%
Zhang [15]	2023	435 CT exams	CT	nnU-Net, 3D CNN	Segmenting and grading sacroiliitis in AS	Dice coefficients: 0.915, 0.889
Lee [31]	2023	492 patients	X-ray	DenseNet121 CNN	Diagnosing sacroiliitis	Sensitivities and specificities peaking at 100%
Berghe [32]	2023	145 patients	CT	U-Net, CNNs for erosion and ankylosis detection	Detecting structural lesions of sacroiliitis	Dice coefficient for segmentation: 0.75
Li [7]	2023	6436 PXRs	Pelvic radiographs (X-ray)	Ensemble deep learning models	Diagnosing and predicting ankylosing spondylitis	Precision: 0.91, Recall: 0.90, AUC: 0.96
Tas [20]	2023	527 patients	MRI	DenseNet201 with GAP layer and KNN classifier	Diagnosing ankylosing spondylitis	F1-scores: 99.80–99.45%
Ureten [19]	2023	585 radiographs	Pelvic radiographs (X-ray)	CNNs with transfer learning	Diagnosing sacroiliitis	Accuracy: 89.9%, Sensitivity: 90.9%, Specificity: 88.9%
Folle [18]	2022	649 patients	MRI	ResNet neural networks	Differentiation of arthritis types	AUC: 0.67–0.75
Bressem [38]	2022	593 patients	MRI	3D U-Net, ResNet-101	Detecting axSpA changes in sacroiliac joint	Sensitivity: 88%, Specificity: 71%
Koo [37]	2022	1280/10,328 radiographs	Digital radiography (X-ray)	Modified HRNet, ResNet 152	Grading of vertebral bodies in AS	Sensitivity: 0.93652, Specificity: 0.97266
Fernandez [6]	2022	267/534 radiographs	Conventional radiographs (X-ray)	CNN-XGBoost model	Classification of sacroiliitis grade	Accuracy: 57%, Sensitivity for all classes except Class 1: over 60%
Gou [34]	2021	100 subjects	MRI	LHR-Net, ResNet-50-based classification network	Segmentation and grading of AS lesions	DSC: 0.71
Lee [33]	2021	60/815 MRI images	MRI	ResNet18-based CNN	Detecting bone marrow edema	Accuracy: 93.55%, Recall: 92.87%
Bressem [8]	2021	1553/458 radiographs	Conventional radiography	ResNet-50 CNN	Detection of radiographic sacroiliitis	AUC: 0.97, Sensitivity: 88%, Specificity: 95%
Rzecki [28]	2021	30 MRI exams	MRI	U-Net-like architecture, VGG-like networks	Detecting bone marrow edema lesions	Sensitivity: 0.88, Specificity: 0.91
Faleiros [26]	2020	56 MRI exams	MRI	SVM, MLP, Instance-Based Algorithm	Classifying active inflammatory sacroiliitis	Sensitivity: 100%, Specificity: 95.6%
Shenkman [30]	2019	242 CT scans	CT	U-Net classifier, random forest	Diagnosing sacroiliitis	Sensitivity: 95%, AUC: 0.97

*MRI* magnetic resonance imaging, *CT* computed tomography, *CNN* convolutional neural network, *SVM* support vector machine, *MLP* multilayer perceptron, *AS* ankylosing spondylitis, *axSpA* axial spondyloarthritis, *PXRs* pelvic radiographs, *GAP* global average pooling, *KNN* k-nearest neighbors, *AUC* area under the curve, *MCC* Matthews correlation coefficient, *DSC* dice similarity coefficient

The radiographic images analyzed in this review included X-ray, MRI, and CT Specifically, MRI was employed in twelve studies, with a focus on the sacroiliac joint and the whole spine. Five studies utilized CT, while another five employed X-ray, primarily for diagnosing sacroiliitis and grading vertebral changes.

The performance of these models was generally promising, though varied. Sensitivity, specificity, and AUROC (Area Under the Receiver Operating Characteristic Curve) are commonly used metrics. For example, Lee et al reported a patient-level sensitivity of 94.7% and specificity of 69.1%, with an AUC of 0.816 [22]. Bressem et al achieved an AUC of 0.94 for detecting inflammatory changes [45]. Koo et al demonstrated high sensitivity 93.6% and accuracy 95.7% for grading vertebral changes in ankylosing spondylitis [44].

Most of the studies did not have external validation, with only four studies that incorporated some form of external validation. Bordner used an external ASAS cohort of 47 patients [43]. Roles employed an independent test set of 243 SpA patient MRI scans [9]. Bressem (2021) utilized a second cohort of 458 radiographs for validation [11]. Li conducted a multicenter study, which provides a degree of external validation [10].

#### Clinical tasks

**Fig. 4:**
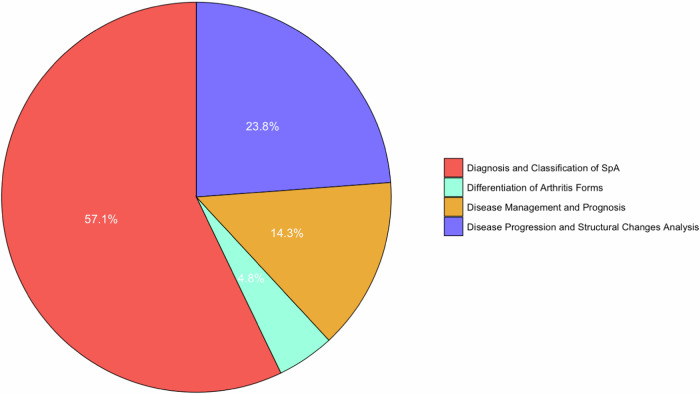
Distribution of the clinical tasks performed by the deep learning models

The clinical tasks of the included studies can be grouped into four broad categories: Diagnosis and Classification of SpA, Differentiation of Arthritis Forms, Disease Progression and Structural Changes Analysis, and Disease Management and Prognosis (Fig. 4 visually illustrates the distribution of the clinical tasks among the included studies). Each category encapsulates the key findings, performance metrics, and notable characteristics of the AI models utilized in the studies.

### Diagnosis and classification of SpA

In MRI studies, Lee et al (2023—MRI), Bressem et al (2022—MRI), Bordner et al, and Roles et al focused on diagnosing axial SpA [22, 42, 43, 45]. Lee et al utilized a two-stage faster R-CNN and VGG-19 network achieving a sensitivity of 94.7% and specificity of 69.1% [22]. Bressem et al (2022) reported an AUC of 0.94 with their 3D dual-encoder ResNet-101 for detecting inflammatory changes [45]. Bordner et al developed a mask-RCNN model predicting active sacroiliitis with high accuracy, evidenced by MCC values up to 0.90 and AUCs up to 0.98 [36]. Roles et al study utilized a ResNet18-based CNN, achieving a high cross-validation AUC of 94.5% [42]. Zhang (2024) et al and Li et al demonstrated the potential of CNNs in diagnosing sacroiliitis and ankylosing spondylitis [9, 20], with Li’s ensemble models surpassing human experts (precision, recall, and AUC values of 0.91, 0.90, and 0.96, respectively) [10]. Faleiros et al and Lin et al also showed high accuracy in MRI classification for active inflammatory sacroiliitis and spinal inflammation, respectively, matching radiologist performance [32, 33].

In X-ray studies, Lee et al (2023—X-ray) and Bressem et al (2021—X-ray) utilized deep learning models to diagnose sacroiliitis from radiographs [11, 38]. Lee et al utilized a DenseNet121 CNN, achieving up to 100% sensitivity and specificity for certain grades of spondylolisthesis [38]. Bressem et al (2021), employedResNet-50, with AUC of 0.97 and 0.94 invalidation and test datasets, respectively [11]. Additionally, Ureten et al applied pre-trained models like VGG-16, ResNet-101, and Inception-v3, reaching an accuracy of 89.9% with VGG-16 [24].

For CT images, Zhang et al (2023) utilized nnU-Net and a 3D CNN to grade sacroiliitis in ankylosing spondylitis [20]. The nnU-Net achieved dice coefficient of 0.889 for the test set, indicating high segmentation accuracy. For grading sacroiliitis, the 3D CNN model yielded micro average AUCs of 0.91 for the test set, with accuracy levels of 0.802.

### Differentiation of arthritis forms

Folle et al study on differentiating psoriatic arthritis (PsA) from other forms using ResNet neural networks showed AUC values ranging from 67 to 75% [23].

### Disease progression and structural changes analysis

Five studies examined radiographic progression and structural changes. Koo et al study utilized a CNN model for grading vertebral bodies changes, showing high sensitivity and accuracy [44]. Gou et al used LHR-Net for lesion segmentation and ankylosing spondylitis grading, with a dice coefficient score of 0.71 [41].

Zhang (2023) et al and Berghe et al leveraged CT imaging for sacroiliitis analysis [20, 39]. Zhang et al nnU-Net achieved a dice coefficient of 0.915, indicating high segmentation accuracy [20]. Berghe et al study utilized U-Net for sacroiliac joint segmentation with a dice coefficient of 0.75 [39]. Lee et al (2021) utilized ResNet18 to detect bone marrow edema in the sacroiliac joints from MRI images, achieving an impressive accuracy of 93.5% [40].

### Disease management and prognosis

Three studies explored deep learning applications beyond diagnosis in SpA. Koo et al developed a deep learning model for grading vertebral body corners to assess radiographic progression in ankylosing spondylitis patients [44]. This model showed high sensitivity (0.93652) and accuracy (0.95743) for grade 3 changes, aiding in disease monitoring [44]. Li et al created an AI tool that not only diagnosed ankylosing spondylitis but also predicted disease course, achieving high precision (0.91) and recall (0.90) [10]. Additionally, Rzecki et al (2021) demonstrated an automated method for quantifying bone marrow edema lesion volumes in axial spondyloarthritis, with a high correlation (0.866) to manual assessments [35].

## Discussion

We analyzed 21 studies that employed deep learning models to enhance the diagnostic imaging of SpA using MRI, CT, and X-ray techniques. Our findings may indicate MRI as the most effective modality, highlighted by Lee et al’s two-stage framework with high sensitivity and specificity [22], and Bressem et al’s 3D CNN architecture, reaching an AUC of 0.94 [45]. These models often surpassed the performance of human experts in diagnosing and classifying axial SpA. CT imaging also demonstrated strong results, especially in segmentation and grading of sacroiliitis, with Zhang et al nnU-Net showing high segmentation accuracy and diagnostic reliability [20]. In contrast, X-ray-based models, while still effective, generally showed lower performance compared to MRI and CT. However, it is important to note that many of the reviewed studies are largely proof-of-concept and not directly comparable.

At this stage, we cannot provide a precise roadmap for the future development of these models. More work is needed for validation and fine-tuning. However, our results indicate that deep learning in MRI diagnosis of SpA, and also maybe in aiding management and predicting prognosis, is promising and could be integrated into the daily work of radiologists in the near future. Currently, to the best of our knowledge, there are no commercial deep learning applications specifically designed for SpA available in clinical practice. This gap may result from regulatory requirements and the variability in disease presentation necessitates further validation to ensure tools are robust and reliable across diverse patient populations.

The integration of CNNs and U-Net has markedly improved the accuracy of imaging diagnoses in SpA, presenting a potential in transformation rheumatology and radiology [11, 32–34, 36]. Deep learning methods have excelled in identifying complex patterns and segmenting medical images [6, 18, 21]. However, the performance metrics across studies, such as sensitivity, specificity, and AUC, exhibit variability. This variability underscores the necessity for standardized benchmarks in future research to ensure the consistency and reliability of AI applications.

Integrating AI with human expertise is crucial for nuanced interpretation and decision-making [6, 18]. For example, the study by Koo et al demonstrated high sensitivity and accuracy in grading vertebral changes in ankylosing spondylitis using a modified HRNet alongside a ResNet 152-based CNN [44]. This integration highlights AI’s potential to augment human diagnostic capabilities, emphasizing the importance of collaborative efforts between technology and clinical expertise [6, 7, 27, 46]. Although current evidence shows promising results for integrating deep learning, especially in MRI and CT [6, 18, 21], practical implementation in real-world clinical scenarios requires further assessment and testing. Deep learning has been incorporated into many products by major tech companies such as Apple, Google, Microsoft, and Facebook [47]. While AI has been widely used in other fields, its application in medicine has only recently gained momentum [47]. Introducing these models into clinical practice will certainly involve a learning curve and should be trial-tested and validated. Additionally, attention U-Net algorithm provided a unique adaptation by enhancing the standard U-Net with attention mechanisms for more effective focus on pertinent image areas [26]. With the recent advent of attention and transformer models in computer vision, their utilization for SpA is expected to increase in the coming years.

There is an established delay in diagnosing SpA due to its diverse clinical presentation and the challenge of identifying it by healthcare providers [3, 4, 48]. Additionally, radiographic identification is sometimes difficult, especially in early disease stages [2, 4]. These challenges suggest that deep learning methods could soon be used to assist radiologists, ease their workload, and potentially reduce diagnostic delays, benefiting both patients and health systems.

The results of our study, along with the current direction of the literature, underscore the possible potential impact of AI’s integration into clinical practice, particularly within the fields of rheumatology and radiology [6–8, 49, 50]. However, prospective studies are crucial to validate AI models, especially those based on deep learning, and to investigate their applicability in the clinical setting [51]. The use of AI in diagnostic imaging faces challenges. These include the dependency on data quality and variability in study methodologies [6, 27].

Importantly, it is essential to consider the role of human-AI interaction. AI systems, while powerful, are not replacements for human expertise but tools to enhance diagnostic capabilities. Future development should focus on integrating AI models with human oversight, ensuring that the models are interpretable and clinically validated. A structured roadmap, including large-scale prospective studies and regulatory approvals, is crucial for transitioning from proof-of-concept to routine clinical application.

The limitations of the reviewed studies stem from their retrospective nature, often leading to challenges in data diversity and applicability in clinical settings. Additionally, most studies did not compare AI model performance with human practitioners. For instance, Lee et al relied on expert consensus for ground truth, possibly limiting real-world applicability [22]. Folle et al faced challenges with insufficient training datasets, a common issue in deep learning studies that affects model robustness [23]. Similarly, Bressem et al encountered biases due to their selective use of imaging techniques, potentially affecting the comprehensiveness of their findings [45]. The reviewed studies did not directly assess the clinical and cost benefits of AI-based technology in SpA diagnosis. They also did not fully address potential risks, such as false positive or negative results. The exact impact on patient management and treatment decisions remains unclear. Although results show high precision and potential as an augmentation tool for radiologists, future studies should evaluate these specific aspects to fully understand AI’s role in clinical practice, using larger datasets and prospective designs. These examples highlight common issues in this specific area of AI research, such as data quality, model validation, and potential biases, which are important for the practical application of AI in medical imaging (Table 2).

**Table 2:** Key findings, implications and challenges of the included studies

Author	Year	Main findings	Implications	Challenges
Lin [27]	2024	AI model shows comparable sensitivity and specificity to radiologist in identifying spinal inflammation in axSpA.	Indicates potential of AI in improving MRI interpretation for axial spondyloarthritis, aiding clinical management and diagnosis.	Potential bias in establishing ground-truth masks, small sample size, focus on identification over precise outlining
Zhang [9]	2024	Excellent diagnostic performance in diagnosing axSpA-related sacroiliitis using CNNs and ensemble models	Shows robust potential of DLR combined with clinical factors in diagnosing sacroiliitis, advancing AI's role in radiographic diagnosis	Limited to oblique coronal MRI images
Lee [17]	2023	Improved detection of inflammatory sacroiliitis using a two-stage AI model	Potential in enhancing MRI accuracy and reducing inter-observer variability in clinical settings	Reliance on expert consensus for ground truth, lack of real-world testing
Zhang [15]	2023	High segmentation accuracy and reliable grading of sacroiliitis on CT images using nnU-Net and 3D CNN	Demonstrates potential of deep learning for automated segmentation and grading of sacroiliitis in AS	No gold standard for sacroiliitis diagnosis, small dataset with potential bias
Lee [31]	2023	High accuracy in detecting and grading sacroiliitis using DenseNet121 CNN on X-ray images	Diagnosing SpA, impacting treatment strategies and patient outcomes	Variability in image quality, patient positioning
Berghe [32]	2023	Effective detection of structural lesions of sacroiliitis on pelvic CT scans using U-Net and CNNs	Suggests AI’s significant role in diagnosing sacroiliitis, leading to earlier detection and treatment, and integrated diagnosis with clinical data	Limited number of pelvic CTs, focus on tertiary university hospitals
Li [7]	2023	Superior diagnostic capabilities of ensemble DL models in diagnosing ankylosing spondylitis, effective even with smartphone-captured images.	Suggests AI can enhance the diagnostic process for ankylosing spondylitis, especially in areas with limited specialized care.	Difficulty in amassing large-scale PXR dataset
Tas [20]	2023	High accuracy, recall, precision, and F1-scores in diagnosing ankylosing spondylitis using DenseNet201 with GAP layer and KNN	Implies AI models like ASNET could improve ankylosing spondylitis diagnosis accuracy and healthcare resource efficiency.	Lack of external validation
Ureten [19]	2023	Promising results from CNN models in diagnosing sacroiliitis from pelvic radiographs, with VGG-16 showing slight superiority	Demonstrates potential of deep learning methods in aiding sacroiliitis diagnosis, possibly reducing reliance on advanced imaging like MRI.	Manual cropping of images, limited dataset size, lack of classification according to modified New York criteria
Folle [18]	2022	AI effectively differentiates between PsA, seropositive RA, and seronegative RA using MRI of the hand	Potential in differential diagnosis of arthritis forms using MRI scans	The need for larger training datasets
Bressem [38]	2022	High sensitivity and specificity for detecting axSpA changes in sacroiliac joint with 3D U-Net and ResNet-101	Potential in aiding axial spondyloarthritis diagnosis, especially in distinguishing between inflammatory and structural changes	Potential biases due to exclusive use of semicoronal images
Koo [37]	2022	High performance in grading vertebral bodies, aiding in the detection of mSASSS in AS patients	Suggests deep learning’s role in enhancing radiographic assessment of ankylosing spondylitis	Exclusion of cases with severe malformations, risk of overfitting, lack of clinical verification
Fernandez [6]	2022	AI model effectively detects the grade of sacroiliitis on conventional radiographs with over 60% accuracy.	Highlights AI’s aid in detecting radiographic sacroiliitis, assisting non-expert clinicians in diagnosis and reducing delays.	Complex dataset, small number of images per class, complete misclassification of Class 1
Gou [34]	2021	LHR-Net demonstrated superior lesion segmentation and grading performance in AS diagnosis	Highlights AI’s significant potential in enhancing radiographic diagnosis of SpA	Data variability, generalizability across different medical imaging scenarios
Lee [33]	2021	High accuracy in detecting bone marrow edema from MRI images using ResNet-based CNN	Demonstrates AI’s effectiveness in aiding axial spondyloarthritis diagnosis, leading to improved detection and management	Lack of external validation, potential for selection bias
Bressem [8]	2021	Near expert-level detection of radiographic sacroiliitis with high agreement with human readers	Supports potential use of AI for accurate sacroiliitis detection, beneficial in various clinical settings	Patients already diagnosed with axSpA, unknown performance in undiagnosed patients
Rzecki [28]	2021	High precision in automated detection and volume assessment of inflammatory lesions in axial spondyloarthritis	Suggests significant potential for AI in enhancing diagnosis accuracy and efficiency, especially in the early detection of bone marrow edema lesions	Challenges in training deep learning models to distinguish closely located bones, reliance on expert knowledge for manual segmentation
Faleiros [26]	2020	Machine learning methods, particularly MLP, accurately classify active inflammatory sacroiliitis in MRI images	Suggests AI can significantly aid in diagnosing active inflammatory sacroiliitis, improving early detection and treatment.	Small sample, segmentation by one radiologist, manual selection of images
Shenkman [30]	2019	AI model accurately detects and grades sacroiliitis, assisting radiologists in more efficient diagnosis	Enhances diagnostic processes in radiology, indicating AI’s role in improving efficiency and accuracy	Potential for selection bias

*AI* artificial intelligence, *axSpA* axial spondyloarthritis, *AS* ankylosing spondylitis, *MRI* magnetic resonance imaging, *CT* computed tomography, *CNN* convolutional neural network, *MLP* multilayer perceptron, *PsA* psoriatic arthritis, *RA* rheumatoid arthritis, *mSASSS* Modified Stoke Ankylosing Spondylitis Spinal Score, *LHR-Net* lightweight hybrid multi-scale convolutional neural network, *DLR* deep learning radiomics, *nnU-Net* no-new-UNet, *GAP* global average pooling, *kNN* k-nearest neighbors, *PXRs* pelvic radiographs

Additionally, a meta-analysis was not conducted due to the heterogeneity of the studies involved [52].

## Conclusions

Deep learning models show promise in improving the accuracy of SpA imaging diagnostics across various modalities, including MRI and CT. These models have the potential to reduce diagnostic delays, but the small sample sizes and proof-of-concept nature of many studies emphasize the need for larger, more diverse datasets and further prospective validation. Continued development and real-world testing are necessary to ensure the generalizability and clinical integration of these AI models.

## Supplementary information

ELECTRONIC SUPPLEMENTARY MATERIAL

## Abbreviations

AI: Artificial intelligence
AS: Ankylosing spondylitis
AUC: Area under the receiver operating characteristic curve
CNN: Convolutional neural network
CT: Computed tomography
HRNet: High-resolution network
MCC: Matthews Correlation Coefficient
MRI: Magnetic resonance imaging
PRISMA: Preferred Reporting Items for Systematic Reviews and Meta-Analyses
PROSPERO: International Prospective Register of Systematic Reviews
QUADAS-2: Quality Assessment of Diagnostic Accuracy Studies-2
SpA: Spondyloarthropathies
U-Net: A type of convolutional neural network used for image segmentation
VGG: Visual Geometry Group (a type of convolutional neural network architecture)

## References

[1] Fragoulis GE, Liava C, Daoussis D, Akriviadis E, Garyfallos A, Dimitroulas T (2019) Inflammatory bowel diseases and spondyloarthropathies: from pathogenesis to treatment. World J Gastroenterol 25:2162–2176 31143068 10.3748/wjg.v25.i18.2162 PMC6526158

[2] Poddubnyy D (2020) Classification vs diagnostic criteria: the challenge of diagnosing axial spondyloarthritis. Rheumatology 59:iv6–iv17 33053191 10.1093/rheumatology/keaa250 PMC7566535

[3] Zhao SS, Pittam B, Harrison NL, Ahmed AE, Goodson NJ, Hughes DM (2021) Diagnostic delay in axial spondyloarthritis: a systematic review and meta-analysis. Rheumatology 60:1620–1628 33428758 10.1093/rheumatology/keaa807

[4] Weber U, Maksymowych WP (2013) Advances and challenges in spondyloarthritis imaging for diagnosis and assessment of disease. Curr Rheumatol Rep 15:345 23771558 10.1007/s11926-013-0345-z

[5] Grigoryan M, Roemer FW, Mohr A, Genant HK (2004) Imaging in spondyloarthropathies. Curr Rheumatol Rep 6:102–109 15016340 10.1007/s11926-004-0054-8

[6] Hosny A, Parmar C, Quackenbush J, Schwartz LH, Aerts HJWL (2018) Artificial intelligence in radiology. Nat Rev Cancer 18:500–510 29777175 10.1038/s41568-018-0016-5 PMC6268174

[7] McMaster C, Bird A, Liew DFL et al (2022) Artificial intelligence and deep learning for rheumatologists. Arthritis Rheumatol 74:1893–1905 35857865 10.1002/art.42296 PMC10092842

[8] Adams LC, Bressem KK, Ziegeler K, Vahldiek JL, Poddubnyy D (2024) Artificial intelligence to analyze magnetic resonance imaging in rheumatology. Joint Bone Spine 91:105651 37797827 10.1016/j.jbspin.2023.105651

[9] Soffer S, Ben-Cohen A, Shimon O, Amitai MM, Greenspan H, Klang E (2019) Convolutional neural networks for radiologic images: a radiologist’s guide. Radiology 290:590–606 30694159 10.1148/radiol.2018180547

[10] Li H, Tao X, Liang T et al (2023) Comprehensive AI-assisted tool for ankylosing spondylitis based on multicenter research outperforms human experts. Front Public Health 11:1063633 10.3389/fpubh.2023.1063633 PMC9947660 36844823

[11] Bressem KK, Vahldiek JL, Adams L et al (2021) Deep learning for detection of radiographic sacroiliitis: achieving expert-level performance. Arthritis Res Ther 23:106 33832519 10.1186/s13075-021-02484-0 PMC8028815

[12] Zhang K, Liu C, Pan J et al (2024) Use of MRI-based deep learning radiomics to diagnose sacroiliitis related to axial spondyloarthritis. Eur J Radiol 172:111347 38325189 10.1016/j.ejrad.2024.111347

[13] Klang E (2018) Deep learning and medical imaging. J Thorac Dis 10:1325–1328 29708147 10.21037/jtd.2018.02.76 PMC5906243

[14] Barash Y, Klang E (2019) Automated quantitative assessment of oncological disease progression using deep learning. Ann Transl Med 7:S379–S379 32016097 10.21037/atm.2019.12.101 PMC6976497

[15] Esteva A, Chou K, Yeung S et al (2021) Deep learning-enabled medical computer vision. NPJ Digit Med 4:5 33420381 10.1038/s41746-020-00376-2 PMC7794558

[16] Li J, Jiang P, An Q, Wang GG, Kong HF (2024) Medical image identification methods: a review. Comput Biol Med 169:107777 38104516 10.1016/j.compbiomed.2023.107777

[17] Chen X, Wang X, Zhang K et al (2022) Recent advances and clinical applications of deep learning in medical image analysis. Med Image Anal 79:102444 35472844 10.1016/j.media.2022.102444 PMC9156578

[18] Yamashita R, Nishio M, Do RKG, Togashi K (2018) Convolutional neural networks: an overview and application in radiology. Insights Imaging 9:611–629 29934920 10.1007/s13244-018-0639-9 PMC6108980

[19] Zhou Z, Rahman Siddiquee MM, Tajbakhsh N, Liang J (2018) UNet++: a nested U-Net architecture for medical image segmentation. Deep Learn Med Image Anal Multimodal Learn Clin Decis Support (2018) 11045:3–11 10.1007/978-3-030-00889-5_1 PMC7329239 32613207

[20] Zhang K, Luo G, Li W et al (2023) Automatic image segmentation and grading diagnosis of sacroiliitis associated with AS using a deep convolutional neural network on CT images. J Digit Imaging 36:2025–2034 37268841 10.1007/s10278-023-00858-1 PMC10501961

[21] Xu W, Fu YL, Zhu D (2023) ResNet and its application to medical image processing: research progress and challenges. Comput Methods Prog Biomed 240:107660 10.1016/j.cmpb.2023.107660 37320940

[22] Lee S, Jeon U, Lee JH et al (2023) Artificial intelligence for the detection of sacroiliitis on magnetic resonance imaging in patients with axial spondyloarthritis. Front Immunol 14:1278247 10.3389/fimmu.2023.1278247 PMC10676202 38022576

[23] Folle L, Bayat S, Kleyer A et al (2022) Advanced neural networks for classification of MRI in psoriatic arthritis, seronegative, and seropositive rheumatoid arthritis. Rheumatology 61:4945–4951 35333316 10.1093/rheumatology/keac197

[24] Üreten K, Maraş Y, Duran S, Gök K (2023) Deep learning methods in the diagnosis of sacroiliitis from plain pelvic radiographs. Mod Rheumatol 33:202–206 34888699 10.1093/mr/roab124

[25] McBee MP, Awan OA, Colucci AT et al (2018) Deep learning in radiology. Acad Radiol 25:1472–1480 29606338 10.1016/j.acra.2018.02.018

[26] Al Qurri A, Almekkawy M (2023) Improved UNet with attention for medical image segmentation. Sensors (Basel) 23:8589 37896682 10.3390/s23208589 PMC10611347

[27] Najjar R (2023) Redefining radiology: a review of artificial intelligence integration in medical imaging. Diagnostics 13:2760 37685300 10.3390/diagnostics13172760 PMC10487271

[28] Tas NP, Kaya O, Macin G, Tasci B, Dogan S, Tuncer T (2023) ASNET: a novel AI framework for accurate ankylosing spondylitis diagnosis from MRI. Biomedicines 11:2441 37760882 10.3390/biomedicines11092441 PMC10525210

[29] Page MJ, McKenzie JE, Bossuyt PM et al (2021) The PRISMA 2020 statement: an updated guideline for reporting systematic reviews. BMJ 372:n71 10.1136/bmj.n71 PMC8005924 33782057

[30] Schiavo JH (2019) PROSPERO: an international register of systematic review protocols. Med Ref Serv Q 38:171–180 31173570 10.1080/02763869.2019.1588072

[31] Whiting PF (2011) QUADAS-2: a revised tool for the quality assessment of diagnostic accuracy studies. Ann Intern Med 155:529 22007046 10.7326/0003-4819-155-8-201110180-00009

[32] Faleiros MC, Nogueira-Barbosa MH, Dalto VF et al (2020) Machine learning techniques for computer-aided classification of active inflammatory sacroiliitis in magnetic resonance imaging. Adv Rheumatol 60:25 32381053 10.1186/s42358-020-00126-8

[33] Lin Y, Chan SCW, Chung HY, Lee KH, Cao P (2024) A deep neural network for MRI spinal inflammation in axial spondyloarthritis. Eur Spine J 33:4125–4134 10.1007/s00586-023-08099-0 38190004

[34] Fernandez E, Garrigos J, Martinez JJ et al (2022) A new artificial intelligence approach for the radiographic classification of sacroiliitis. Springer, Cham, pp 383–390

[35] Rzecki K, Kucybała I, Gut D et al (2021) Fully automated algorithm for the detection of bone marrow oedema lesions in patients with axial spondyloarthritis—feasibility study. Biocybern Biomed Eng 41:833–853

[36] Tenório APM, Ferreira-Junior JR, Dalto VF et al (2022) Radiomic quantification for MRI assessment of sacroiliac joints of patients with spondyloarthritis. J Digit Imaging 35:29–38 34997373 10.1007/s10278-021-00559-7 PMC8854535

[37] Shenkman Y, Qutteineh B, Joskowicz L et al (2019) Automatic detection and diagnosis of sacroiliitis in CT scans as incidental findings. Med Image Anal 57:165–175 31323597 10.1016/j.media.2019.07.007

[38] Lee KH, Lee RW, Lee KH, Park W, Kwon SR, Lim MJ (2023) The development and validation of an AI diagnostic model for sacroiliitis: a deep-learning approach. Diagnostics 13:3643 38132228 10.3390/diagnostics13243643 PMC10743277

[39] Van Den Berghe T, Babin D, Chen M et al (2023) Neural network algorithm for detection of erosions and ankylosis on CT of the sacroiliac joints: multicentre development and validation of diagnostic accuracy. Eur Radiol 33:8310–8323 37219619 10.1007/s00330-023-09704-y

[40] Lee KH, Choi ST, Lee GY, Ha YJ, Choi SI (2021) Method for diagnosing the bone marrow edema of sacroiliac joint in patients with axial spondyloarthritis using magnetic resonance image analysis based on deep learning. Diagnostics 11:1156 34202607 10.3390/diagnostics11071156 PMC8303557

[41] Gou S, Lu Y, Tong N, Huang L, Liu N, Han Q (2021) Automatic segmentation and grading of ankylosing spondylitis on MR images via lightweight hybrid multi-scale convolutional neural network with reinforcement learning. Phys Med Biol 66:205002 10.1088/1361-6560/ac262a 34517352

[42] Roels J, De Craemer A, Renson T et al (2023) Machine learning pipeline for predicting bone marrow edema along the sacroiliac joints on magnetic resonance imaging. Arthritis Rheumatol 75:2169–2177 37410803 10.1002/art.42650

[43] Bordner A, Aouad T, Medina CL et al (2023) A deep learning model for the diagnosis of sacroiliitis according to Assessment of SpondyloArthritis International Society classification criteria with magnetic resonance imaging. Diagn Inter Imaging 104:373–383 10.1016/j.diii.2023.03.008 37012131

[44] Koo BS, Lee JJ, Jung JW et al (2022) A pilot study on deep learning-based grading of corners of vertebral bodies for assessment of radiographic progression in patients with ankylosing spondylitis. Ther Adv Musculoskelet Dis 14:1759720×2211140 10.1177/1759720X221114097 PMC9310199 35898565

[45] Bressem KK, Adams LC, Proft F et al (2022) Deep learning detects changes indicative of axial spondyloarthritis at MRI of sacroiliac joints. Radiology 305:655–665 35943339 10.1148/radiol.212526

[46] Beam AL, Drazen JM, Kohane IS, Leong TY, Manrai AK, Rubin EJ (2023) Artificial intelligence in medicine. N Engl J Med 388:1220–1221 36988598 10.1056/NEJMe2206291

[47] Lee JG, Jun S, Cho YW et al (2017) Deep learning in medical imaging: general overview. Korean J Radiol 18:570 28670152 10.3348/kjr.2017.18.4.570 PMC5447633

[48] Walsh JA, Magrey M (2021) Clinical manifestations and diagnosis of axial spondyloarthritis. J Clin Rheumatol 27:e547–e560 33105312 10.1097/RHU.0000000000001575 PMC8612900

[49] Chinnadurai S, Mahadevan S, Navaneethakrishnan B, Mamadapur M (2023) Decoding applications of artificial intelligence in rheumatology. Cureus 15:e46164 10.7759/cureus.46164 PMC10613315 37905264

[50] Davenport T, Kalakota R (2019) The potential for artificial intelligence in healthcare. Future Health J 6:94–98 10.7861/futurehosp.6-2-94 PMC6616181 31363513

[51] Rajpurkar P, Chen E, Banerjee O, Topol EJ (2022) AI in health and medicine. Nat Med 28:31–38 35058619 10.1038/s41591-021-01614-0

[52] Higgins JPT, Thompson SG (2002) Quantifying heterogeneity in a meta-analysis. Stat Med 21:1539–1558 12111919 10.1002/sim.1186

