# Supplementary material for: The role of deep learning in diagnostic imaging of spondyloarthropathies: a systematic review
Source: Eur Radiol. 2024 Dec 10;35(6):3661–72. doi: 10.1007/s00330-024-11261-x (PMC12081588; doi:10.1007/s00330-024-11261-x)

The Role of Deep Learning in Diagnostic Imaging of Spondyloarthropathies: A Systematic Review

ELECTRONIC SUPPLEMENTARY MATERIAL

Specific Boolean strings used to screen the different databases:

PubMed

("Artificial Intelligence" OR "AI" OR "Deep Learning" OR "Machine Learning" OR "Neural Networks" OR "Computer Vision" OR "Automated Diagnosis" OR "Algorithm*") AND

("Spondyloarthritis" OR "Ankylosing Spondylitis" OR "Axial Spondyloarthritis" OR "Spinal Arthritis" OR "Spondyloarthropathy" OR "Inflammatory Back Pain") AND

("Radiography" OR "Radiographic Diagnosis" OR "MRI" OR "Magnetic Resonance Imaging" OR "CT" OR "Computed Tomography" OR "X-Ray" OR "X-Ray Imaging" OR "Radiograph" OR "Imaging Techniques" OR "Diagnostic Imaging")

Embase

('artificial intelligence'/exp OR 'AI' OR 'deep learning' OR 'machine learning' OR 'neural networks' OR 'computer vision' OR 'automated diagnosis' OR 'algorithm*') AND

('spondyloarthritis'/exp OR 'ankylosing spondylitis'/exp OR 'axial spondyloarthritis' OR 'spinal arthritis' OR 'spondyloarthropathy' OR 'inflammatory back pain') AND

('radiography'/exp OR 'radiographic diagnosis' OR 'MRI' OR 'magnetic resonance imaging'/exp OR 'CT' OR 'computed tomography'/exp OR 'x-ray'/exp OR 'x-ray imaging' OR 'radiograph' OR 'imaging techniques' OR 'diagnostic imaging')

Web of Science

(TS=("Artificial Intelligence" OR "AI" OR "Deep Learning" OR "Machine Learning" OR "Neural Networks" OR "Computer Vision" OR "Automated Diagnosis" OR "Algorithm*")) AND

(TS=("Spondyloarthritis" OR "Ankylosing Spondylitis" OR "Axial Spondyloarthritis" OR "Spinal Arthritis" OR "Spondyloarthropathy" OR "Inflammatory Back Pain")) AND

(TS=("Radiography" OR "Radiographic Diagnosis" OR "MRI" OR "Magnetic Resonance Imaging" OR "CT" OR "Computed Tomography" OR "X-Ray" OR "X-Ray Imaging" OR "Radiograph" OR "Imaging Techniques" OR "Diagnostic Imaging"))

Scopus

(TITLE-ABS-KEY ("Artificial Intelligence" OR "AI" OR "Deep Learning" OR "Machine Learning" OR "Neural Networks" OR "Computer Vision" OR "Automated Diagnosis" OR "Algorithm*")) AND (TITLE-ABS-KEY ("Spondyloarthritis" OR "Ankylosing Spondylitis" OR "Axial Spondyloarthritis" OR "Spinal Arthritis" OR "Spondyloarthropathy" OR "Inflammatory Back Pain")) AND (TITLE-ABS-KEY ("Radiography" OR "Radiographic Diagnosis" OR "MRI" OR "Magnetic Resonance Imaging" OR "CT" OR "Computed Tomography" OR "X-Ray" OR "X-Ray Imaging" OR "Radiograph" OR "Imaging Techniques" OR "Diagnostic Imaging"))

Table S1: Impact Factor and Quartile Classifications of Journals Publishing Included Studies.

Author	Year	Journal	Impact Factor (IF)	Quartile
Lee	2023	Frontiers in Immunology	7.6	1
Folle	2022	Rheumatology (Oxford)	5.5	1
Bressem	2022	Radiology	29.1	1
Koo	2022	Therapeutic advances in musculoskeletal disease	3.65	1
Bordner	2023	Diagnostic and Interventional Imaging	7.42	1
Roles	2023	Arthritis & rheumatology	15.4	1
Gou	2021	Physics in medicine and biology	4.1	4
Lee	2021	Diagnostics	3.99	3
Zhang	2024	European journal of radiology	4.5	1
Zhang	2023	Journal of Digital Imaging	4.9	1
Lee	2023	Diagnostics	3.99	3
Berghe	2023	European radiology	7	1
Tenorio	2022	Journal of Digital Imaging	4.9	1
Shenkman	2019	Medical image analysis	10.9	1

Bressem	2021	Arthritis Research & Therapy	5.6	1
Li	2023	Frontiers in public health	3.9	2
Tas	2023	Biomedicines	3	1
Ureten	2023	Modern rheumatology	2.86	2
Rzecki	2021	Biocybernetics and Biomedical Engineering	8.6	2
Fernandez	2022	Lecture Notes on computer science (Book Chapter and Conference article).	NA	NA
Lin	2024	European Spine Journal	2.8	1
Faleiros	2020	Advances in Rheumatology	3	3

*Impact factors were extracted from the websites of each corresponding journal. Quartiles were extracted from Scimago.

Additional explanatory figures

Figure S1: Comparative Overview of Deep Learning and Machine Learning.

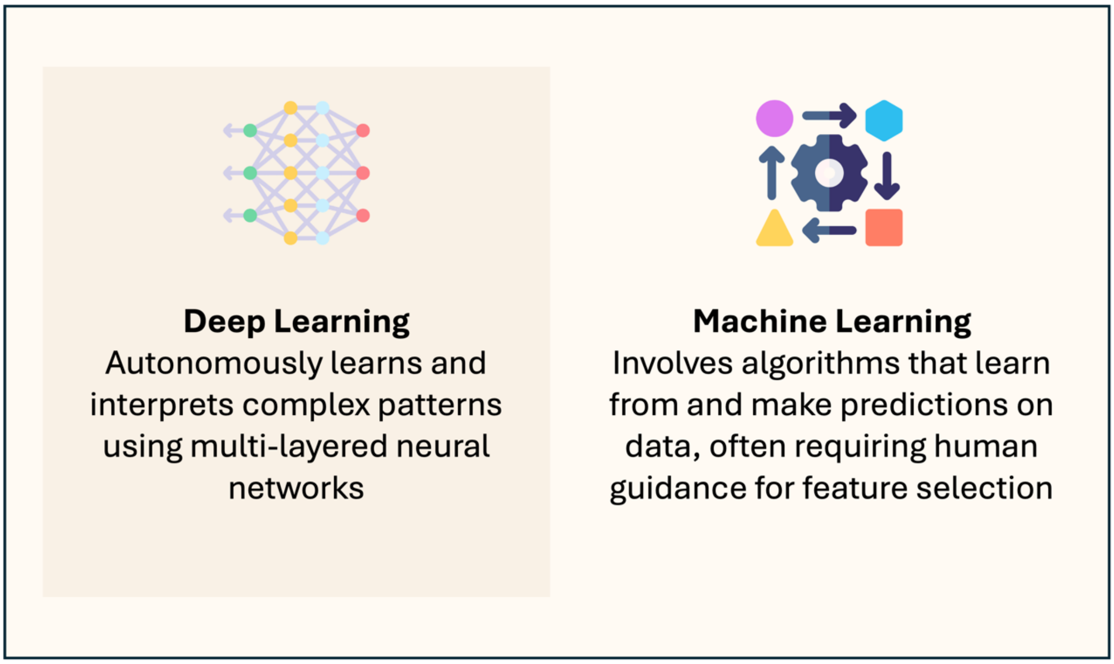

Figure S2: Typical Convolutional Neural Networks (CNN) Workflow for the diagnosis of ankylosis.

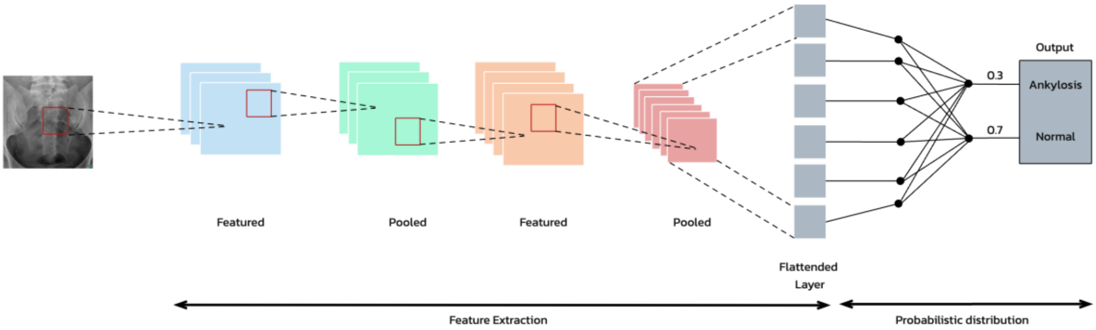

This schematic represents a convolutional neural network (CNN) architecture used for medical image analysis. An MRI input undergoes preprocessing to enhance features, which is then passed through consecutive convolutional layers where features are extracted and analyzed. Each layer applies filters to detect specific attributes, pooling layers to reduce dimensionality, and activation functions to introduce non-linearity, facilitating the identification of complex patterns. The final classification is done through fully connected layers, resulting in the output that categorizes the image into diagnostic categories, such as 'Ankylosis' or 'Normal'.

Figure S3: Deep Learning Analysis of MRI for AS Diagnosis Utilizing 3D-Unet.

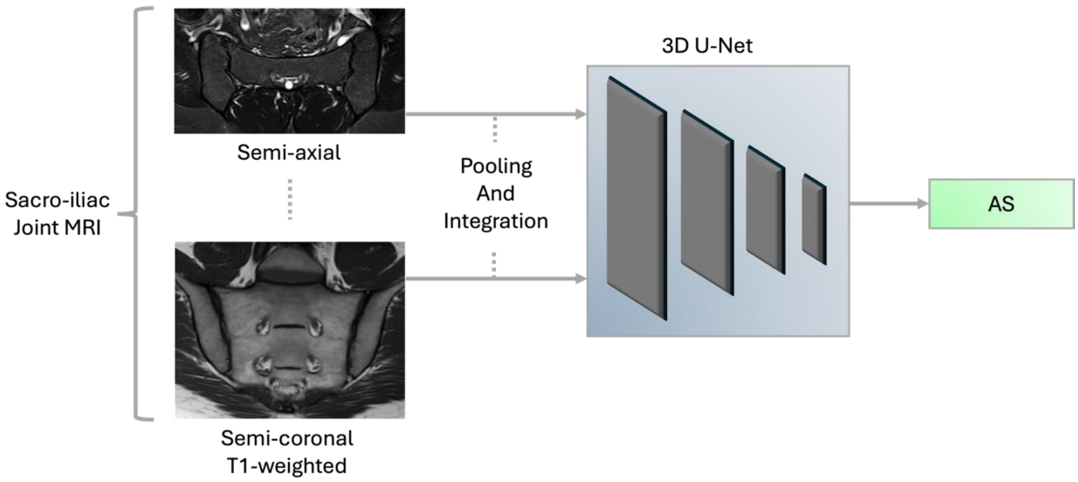

This figure presents a deep learning framework, specifically a U-Net model, processing MRI scans to diagnose Ankylosing Spondylitis (AS). Two MRI inputs, representing different anatomical planes, feed into the neural network which then utilizes convolutional layers to extract features and identify patterns indicative of AS, leading to a diagnostic output.

Figure S4: Distribution of the Included Studies by Quartile and Year.

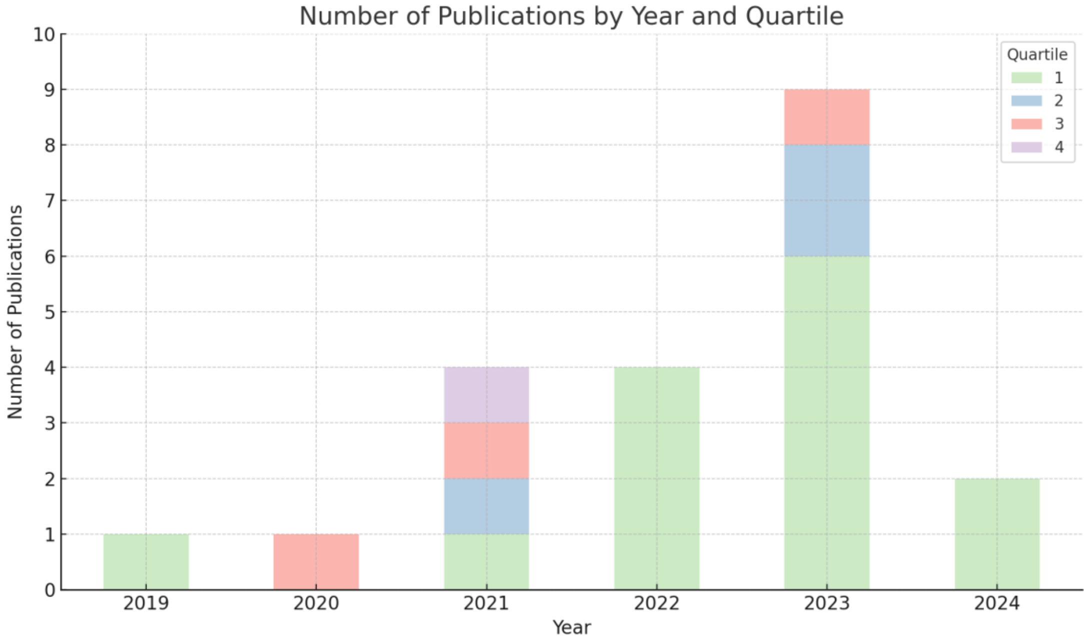

Supplement: Supplementary file 1 — ELECTRONIC SUPPLEMENTARY MATERIAL [file 330_2024_11261_MOESM1_ESM.pdf]
